# Catheter Ablation versus Medical Therapy of Atrial Fibrillation in Patients with Heart Failure: An Updated Systematic Review and Meta-Analysis of Randomized Controlled Trials

**DOI:** 10.3390/jcm11195530

**Published:** 2022-09-21

**Authors:** Michele Magnocavallo, Antonio Parlavecchio, Giampaolo Vetta, Carola Gianni, Marco Polselli, Francesco De Vuono, Luigi Pannone, Sanghamitra Mohanty, Filippo Maria Cauti, Rodolfo Caminiti, Vincenzo Miraglia, Cinzia Monaco, Gian-Battista Chierchia, Pietro Rossi, Luigi Di Biase, Stefano Bianchi, Carlo de Asmundis, Andrea Natale, Domenico Giovanni Della Rocca

**Affiliations:** 1 Arrhythmology Unit, Ospedale San Giovanni Calibita, Fatebefratelli Isola Tiberina, Via Ponte Quattro Capi 39, 00186 Rome, Italy; 2 Cardiology Unit, Department of Clinical and Experimental Medicine, University of Messina, 98122 Messina, Italy; 3 Texas Cardiac Arrhythmia Institute, St. David’s Medical Center, Austin, TX 78705, USA; 4 Department of Medicine, Montefiore Medical Center, Albert Einstein College of Medicine, Bronx, NY 10461, USA; 5 Heart Rhythm Management Centre, Postgraduate Program in Cardiac Electrophysiology and Pacing, Universitair Ziekenhuis Brussel-Vrije Universiteit Brussel, European Reference Networks Guard-Heart, 1090 Brussels, Belgium; 6 Interventional Electrophysiology, Scripps Clinic, La Jolla, CA 92037, USA; 7 Department of Cardiology, MetroHealth Medical Center, Case Western Reserve University School of Medicine, Cleveland, OH 44106, USA

**Keywords:** atrial fibrillation, heart failure, catheter ablation, medical therapy, randomized controlled trials, recurrence

## Abstract

Background: Atrial fibrillation (AF) and heart failure (HF) often coexist and synergistically contribute to an increased risk of hospitalization, stroke, and mortality. Objective: To compare the efficacy of catheter ablation (CA) versus medical therapy (MT) in HF patients with AF. Methods: Electronic databases were queried for randomized controlled trials (RCTs) of CA versus MT of AF in patients with HF. Risk ratios (RRs), mean differences (MDs), and 95% confidence intervals (CIs) were measured using the Mantel–Haenszel method. Results: A total of nine RCTs enrolling 2155 patients met the inclusion criteria. Compared to MT, CA led to a significant reduction in the composite of all-cause mortality and HF hospitalization (24.6% vs. 37.1%; RR: 0.65 (95% CI: 0.53–0.80); *p* < 0.0001), all-cause mortality (8.8% vs. 13.6%; RR: 0.65 (95% CI: 0.51–0.82); *p* = 0.0005), HF hospitalization (15.4% vs. 22.4%; (RR: 0.67 (95% CI: 0.54–0.82); *p* = 0.0001), AF recurrence (31.8% vs. 77.0%; RR: 0.36 (95% CI: 0.24–0.54); *p* < 0.0001), and cardiovascular (CV) death (4.9% vs. 8.4%; RR: 0.58 (95% CI: 0.39–0.86); *p* = 0.007). CA improved the left ventricular ejection fraction (MD:4.76% (95% CI: 2.35–7.18); *p* = 0.0001), 6 min walk test (MD: 20.48 m (95% CI: 10.83–30.14); *p* < 0.0001), peak oxygen consumption (MD: 3.1 2mL/kg/min (95% CI: 1.01–5.22); *p* = 0.004), Minnesota Living with Heart Failure Questionnaire score (MD: −6.98 (95% CI: −12–03, −1.93); *p* = 0.007), and brain natriuretic peptide levels (MD:−133.94 pg/mL (95% CI: −197.33, −70.55); *p* < 0.0001). Conclusions: In HF patients, AF catheter ablation was superior to MT in reducing CV and all-cause mortality. Further significant benefits occurred within the ablation group in terms of HF hospitalizations, AF recurrences, the systolic function, exercise capacity, and quality of life.

## 1. Introduction

Atrial fibrillation (AF) and heart failure (HF) are closely interlinked by pathophysiological mechanisms synergistically contributing to atrial and ventricular myopathy and dysfunction [1,2,3,4].

AF prevalence ranges between <10% and 50%, according to the clinical severity of HF [5]. When AF and HF coexist, their natural history is further complicated by an increased risk of hospitalization, stroke, and all-cause and cardiovascular (CV) mortality [6,7,8]. Therefore, treating AF in HF patients poses several challenges due to the complexity of this population [9,10,11].

As earlier trials have provided conflicting results regarding the best treatment strategy for AF, it is still debated whether rhythm control should be preferred over rate control. Recently, the Early Treatment of Atrial Fibrillation for Stroke Prevention Trial (EAST-AFNET 4) showed the significant benefits of a rhythm control strategy compared to usual care among patients with AF diagnosed within 1 year and other concomitant CV conditions [12].

These results, although encouraging, were not corroborated by two other recent trials [13,14] showing no significant clinical advantage provided by catheter ablation (CA), with both trials being terminated early due to their apparent futility. To explain these uncertainties, we performed a systematic review and meta-analysis of the randomized controlled trials (RCTs), aiming to compare CA versus the medical therapy (MT) of AF in HF patients.

## 2. Methods

### 2.1. Data Sources and Searches

We systematically searched the Medline, Cochrane, Journals@Ovid, and Scopus electronic databases for RCTs published from the time of inception to 30 May 2022 and focusing on CA versus MT in HF patients with AF. Three investigators (A.P, G.V., and M.M.) independently performed searches including the following terms: atrial fibrillation, heart failure, left ventricular dysfunction, and catheter ablation. Detailed information of our literature search strategy is available in the expanded methods.

### 2.2. Study Selection and Outcomes

The preferred reporting items for systematic reviews and meta-analyses (PRISMA) statement for reporting systematic reviews and meta-analyses was used in this study [15].

All titles and full-text versions of the selected relevant RCTs were screened to identify those comparing AF ablation with rate or rhythm control therapy in HF patients, which had at least a 6-month follow-up period, included adults aged 18 years or older, and reported 1 or more clinical outcomes.

Observational studies, nonrandomized trials, editorials, case reports, reviews, expert opinions, and non-English studies were excluded.

### 2.3. Data Extraction and Quality Appraisal

Three investigators (A.P., G.V., and M.M.) extracted data from each study using standardized protocols and reporting forms and independently assessed the quality items. Disagreements were resolved by consensus. The quality of the individual studies was assessed by three investigators (A.P, G.V., and M.M.) using the Cochrane risk of bias Tool [16], as reported in the Supplementary Materials, Figure S1.

### 2.4. Study Endpoints

The primary endpoint was a composite of all-cause mortality and HF hospitalization. These endpoints were also assessed independently. Other secondary endpoints were CV death, AF recurrence rate, changes in the left ventricular ejection fraction [(LVEF) ΔLVEF], changes in quality of life (assessed via the Minnesota Living with Heart Failure Questionnaire (ΔMLHFQ)), changes in peak oxygen consumption (ΔVO_2_max), changes in distance walked during a 6-min walk test (Δ6MWT), and changes in brain natriuretic peptide (ΔBNP) levels.

The safety endpoints were CA-related periprocedural adverse events (e.g., access site complications (femoral bleeding or hematoma), pericardial complications (with and without tamponade), pulmonary vein stenosis, and procedural stroke) and antiarrhythmic drug therapy-related side effects (e.g., proarrhythmic effects, pulmonary, liver, and thyroid toxicity).

### 2.5. Statistical Analysis

Descriptive statistics are presented as means and standard deviations (SD) for the continuous variables or a number of cases (n) and percentages (%) for the dichotomous and categorical variables. The Mantel–Haenszel risk ratio (RR) model was used to summarize the data among the treatment arms. Summary estimates and 95% confidence intervals (CI) were reported for the continuous variables as the standardized mean difference. Freeman–Tukey double arcsine transformation was used to establish the variance of raw proportions. We used the Hartung–Knapp–Sidik–Jonkman method with the random effect model to combine the transformed proportions. The heterogeneity across studies was evaluated by using the Chi^2^, Tau^2^, and Higgins-I^2^ statistics, while random effects models of DerSimonian and Laird or fixed effects models were used in cases of *I*^2^ > 25% or ≤25%, respectively. Sensitivity analyses comparing CA and drug therapy were performed including patients with reduced LVEF (<50%) or persistent AF or with a pharmacological rhythm control strategy. The publication bias was assessed using the funnel plot and Egger’s test. The statistical analysis was performed using Review Manager (RevMan) (computer program) Version 5.4.1, Copenhagen, Denmark: Nordic Cochrane Centre, the Cochrane Collaboration, 2020.

## 3. Results

### 3.1. Study Selection

**Figure 1:**
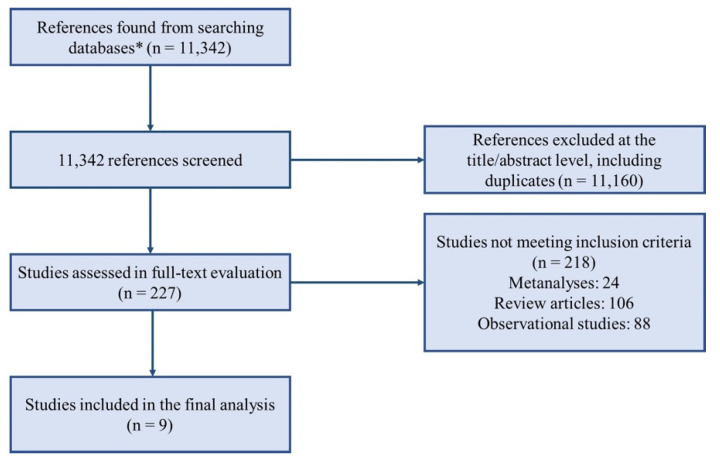
Evidence search and selection of the preferred reporting items for systematic reviews and meta-analyses (PRISMA). * Medline, Cochrane, Journals@Ovid, Scopus.

We screened 11,342 articles, from which 229 full texts were retrieved and reviewed for possible inclusion. A total of nine RCTs [13,14,17,18,19,20,21,22,23,24] comprising 2155 patients were identified (Figure 1).

### 3.2. Baseline Characteristics

Baseline clinical characteristics are reported in Table 1. The nine RCTs enrolled 2155 patients. Among them, 1077 were assigned to CA and 1078 to drug therapy. Overall, 69.9% (*n* = 1507) of patients were male, with an average age of 63.5 years (95% CI: 62.1–64.9), and the mean LVEF was 37.9% (95% CI: 32.3–43.6). Further details on the baseline clinical characteristics are reported in Table 1 and Supplementary Materials, Table S1.

Pulmonary vein isolation (PVI) was the mainstay ablation strategy used for all AF patients randomized to CA. Additional ablation outside the pulmonary veins (e.g., left atrial roof, mitral isthmus and/or cavotricuspid isthmus, posterior wall, left atrial appendage, superior vena cava, complex fractionated atrial electrograms) is specified in the Supplementary Materials, Table S2 [4,25,26,27].

Among the patients randomized to MT, a rate control strategy was pursued in five studies [14,20,21,22,23] and rhythm was pursued control therapy in one, while a combined treatment of both strategies was pursued in three trials (the AMICA (atrial fibrillation management in congestive heart failure with ablation) [13], CASTLE-AF [18], and CABANA trials [17] had 38%, 30%, and 29% of patients on rhythm control, respectively).

### 3.3. Composite Endpoint, All-Cause Mortality, HF Hospitalizations

**Figure 2:**
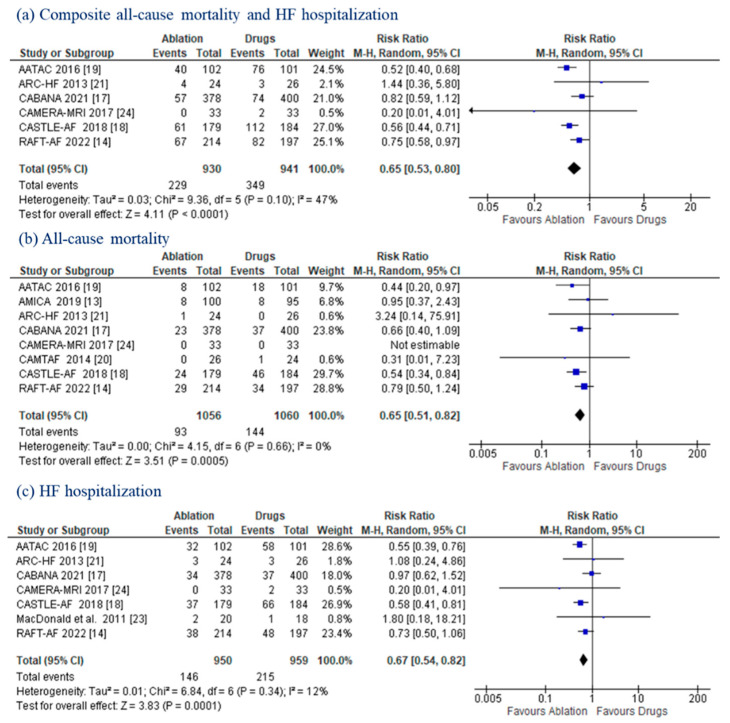
Composite Endpoint, All-Cause Mortality, HF Hospitalizations. Forest plots displaying a decrease in the composite endpoint (**a**), all-cause mortality (**b**), and HF hospitalizations (**c**) in patients with AF and HF undergoing CA versus MT. CI: confidence interval; HF: heart failure.

All trials reported data on all-cause mortality and/or HF hospitalization. AF ablation led to a significant reduction in the composite endpoint (24.6% vs. 37.1%; RR: 0.65 (95% CI: 0.53–0.80); *p* < 0.00001; *I*^2^: 47%]) (Figure 2a). CA also contributed to a significantly lower incidence of all-cause mortality (8.8% vs. 13.6%; RR: 0.65; (95% CI: 0.51–0.82); *p* = 0.0005) and HF hospitalization (15.4% vs. 22.4%; RR: 0.67 (95% CI: 0.54–0.82); *p* = 0.0001) (Figure 2b,c). No statistically significant heterogeneity was documented (*I*^2^ = 0% and 12%, respectively).

### 3.4. Other Secondary Endpoints

**Figure 3:**
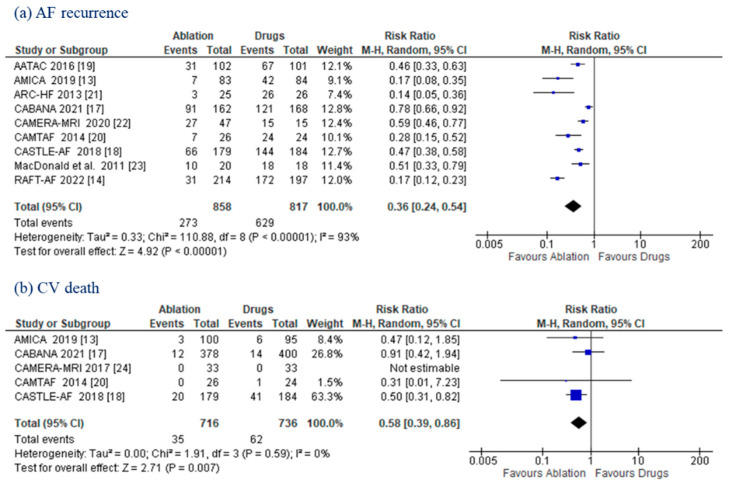
**AF Recurrence and CV Death.** Forest plots displaying risk ratio in AF recurrence (**a**) and cardiovascular death (**b**) between the ablation and drug groups. **AF:** atrial fibrillation; **CI:** confidence interval; **CV:** cardiovascular; **LVEF:** left ventricular ejection fraction.

AF relapse was higher in the MT population (77.0% vs. 31.8%), with CA promoting significantly higher freedom from AF (RR: 0.36; (95% CI: 0.24–0.54); *p* < 0.00001) (Figure 3a). Five RCTs [13,17,18,20,24] reported data on CV death, which was less frequent in the CA group than in the MT group (4.9% vs. 8.4%; RR: 0.58; (95% CI: 0.39–0.86); *p* = 0.007). No heterogeneity (*I*^2^ = 0%) was observed (Figure 3b).

**Figure 4:**
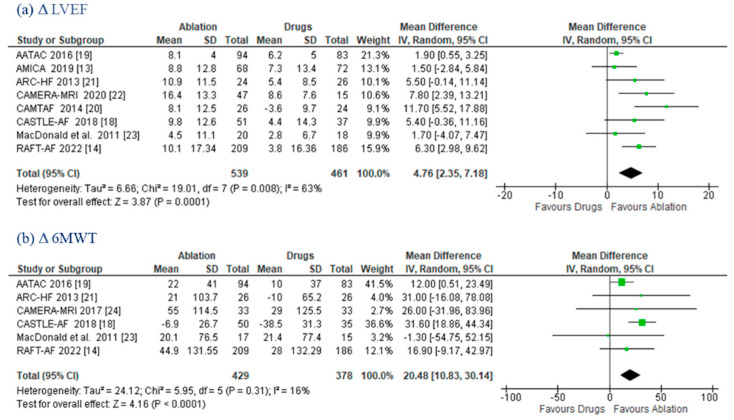
**LVEF and 6MWT.** Forest plots displaying mean differences in LVEF (**a**) and 6MWT (**b**) between the ablation and drug groups: **6MWT:** 6-minute walk test; **CI:** confidence interval; **LVEF:** left ventricular ejection fraction; **SD:** standard deviation.

LVEF changes were evaluated with cardiac ultrasound in five trials [13,14,18,19,20], with magnetic resonance imaging (MRI) in the MacDonald et al. [23] trial, with both MRI and echocardiography in CAMERA-MRI [22], and with radionuclide in the ARC-HF trial [21]. The ablation group had a greater increase in ΔLVEF (MD 4.76%; (95% CI: 2.35–7.18); *p* = 0.0001) compared to MT, with moderate heterogeneity (*I*^2^ = 63%) (Figure 4a).

Δ6MWT data were available for six trials [14,18,19,21,23,24], with AF ablation being associated with a greater improvement at follow-up (MD 20.48 m; (95% CI: 10.83–30.14); *p* < 0.0001) (Figure 4b).

**Figure 5:**
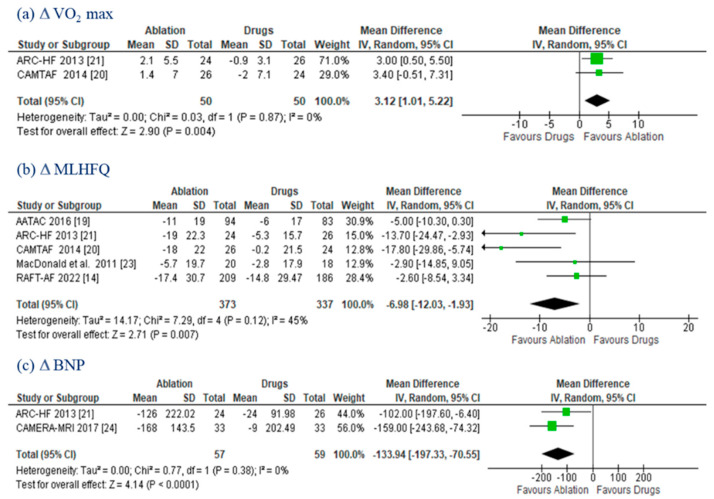
VO_2_ max, MLHFQ, and BNP. Forest plots displaying mean differences in VO_2_ max (**a**), MLHFQ (**b**), and BNP (**c**) between the ablation and drug groups. CI: confidence interval; MLHFQ: Minnesota Living with Heart Failure Questionnaires; SD: standard deviation; VO_2_ Max: peak oxygen consumption.

Data on the ΔVO_2_ max and ΔMLHFQ were provided in two [20,21] and five [14,19,20,21,23] trials, respectively. The improvements promoted by CA were both statistically significant (ΔVO_2_: MD 3.12 mL/kg/min; (95% CI: 1.01–5.22); *p* = 0.004; *I*^2^ = 0%; and ΔMLHFQ: MD −6.98; (95% CI: −12–03, −1.93); *p* = 0.007; *I*^2^ = 45%) (Figure 5a,b). ΔBNP was evaluated in two RCTs [21,24]. CA patients showed a larger reduction (MD −133.94 pg/mL; (95% CI: −197.33, −70.55); *p* < 0.0001; *I*^2^ = 0%) compared to those on drug therapy (Figure 5c).

### 3.5. Safety Endpoints

Data on CA-related adverse events are summarized in Table 2A. Overall, 62 periprocedural adverse events were reported (5.02% (95% CI: 3.44–0.81); Supplementary Materials, Figure S2a). Among them, access site complications were the most common ones (2.37% (95% CI: 1.42–3.5%), followed by pericardial effusion/tamponade in 0.8% (95% CI: 0.23–1.6%).

Anti-arrhythmic drug adverse events are summarized in Table 2B. Only three RCTs (AATAC [19], CABANA [17] and RAFT-AF [14]) reported antiarrhythmic drug-related toxicities, with 31 adverse events being documented (4.28% (95% CI: 2.56–6.39%) Supplementary Materials, Figure S2a).

The stroke risk at follow-up was reported in six RCTs [14,18,20,21,23,24] (Supplementary Materials, Table S3) and was significantly lower after CA compared to MT (0.48% (95% CI: 0–1.61%) vs. 2.60% (95% CI: 1.05–4.62%); *p* = < 0.01)) (Supplementary Materials, Figure S2b).

### 3.6. Sensitivity Analyses

- *Catheter Ablation vs. Rate Control*

**Figure 6:**
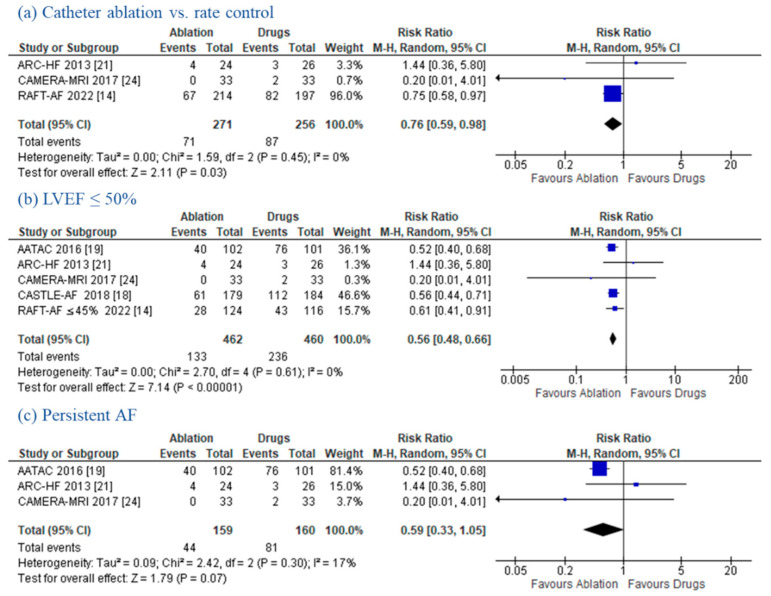
Sensitivity Analysis for the Composite Endpoint. Forest plots displaying a decrease in the composite endpoint in the sensitivity analysis: (**a**) catheter ablation vs. rate control, (**b**) LVEF ≤ 50%, (**c**) persistent AF. CI: confidence interval; HF: heart failure.

Three RCTs [14,21,24] performed a direct comparison between CA and medical rate control only treatment. A sub-analysis of these trials showed that CA was superior to rate control in reducing the composite endpoint of all-cause death and HF hospitalizations (RR 0.76; (95% CI: 0.59–0.98); *p* = 0.03; *I*^2^ = 0%) (Figure 6a). However, no significant differences were found in the all-cause mortality (RR 0.79; (95% CI: 0.51–1.24); *p* = 0.31; *I*^2^ = 0%), HF hospitalizations (RR 0.75; (95% CI: 0.52–1.07); *p* = 0.11; *I*^2^ = 0%), and CV death (RR 0.31; (95% CI: 0.01–7.23); *p* = 0.47). Although significant heterogeneity (I^2^ = 94%) was observed, CA showed a significant reduction in AF recurrence (RR 0.30; (95% CI: 0.14–0.65); *p* = 0.002). Additional data on ΔLVEF, Δ6MWT, and ΔMLFHQ are reported in the Supplementary Materials, Figure S3.

- *LVEF ≤ 50%*

The left ventricular systolic function was not an inclusion criterion in the CABANA [17] and RAFT-AF [14] trials. Therefore, we decided to perform a sensitivity analysis of patients with depressed LVEF (cut-off ≤50% or lower as an inclusion criterion). After CABANA [17] and the subpopulation of patients with LVEF > 45% in the RAFT-AF trial were excluded, CA showed a significant reduction in the composite endpoint (RR 0.56; (95% CI: 0.48–0.66); *p* < 0.00001; *I*^2^ = 0%) (Figure 6b), all-cause mortality (RR 0.57; (95% CI: 0.40–0.81); *p* = 0.002; *I*^2^ = 0%), HF hospitalizations (RR 0.57; (95% CI: 0.45–0.72); *p* < 0.00001; *I*^2^ = 0%), CV death (RR 0.49; (95% CI: 0.31–0.78); *p* = 0.003; *I*^2^ = 0%), and AF recurrence (RR 0.39; (95% CI: 0.29–0.53); *p* < 0.00001; *I*^2^ = 75%). Further secondary endpoint analyses (e.g., ΔLVWEF, Δ6MWT, ΔMLFHQ) are depicted in the Supplementary Materials, Figure S4.

- *Persistent AF*

The CASTLE-AF [18], CABANA [17] and RAFT-AF [14] trials included a mixed population of patients with either paroxysmal or persistent AF. These RCTs were excluded for the purpose of performing a sensitivity analysis focusing on persistent AF patients only (Supplementary Materials, Figure S5). Compared to patients on MT, those undergoing CA showed a trend towards a reduction in the composite endpoint (RR 0.59; (95% CI: 0.33–1.05); *p* = 0.07; *I*^2^ = 17%) (Figure 6c) and a significative reduction in HF hospitalizations (RR 0.57; (95% CI: 0.41–0.78); *p* = 0.0006; *I*^2^ = 0%) and AF recurrence (RR 0.35; (95% CI: 0.22–0.55); *p* < 0.00001; *I*^2^ = 82%). No differences were documented in regard to all-cause mortality (RR 0.63; (95% CI: 0.35–1.12); *p* = 0.11; *I*^2^ = 0%) and CV death (RR 0.44; (95% CI: 0.13–1.54); *p* = 0.20; *I*^2^ = 0%). Other outcome data (e.g., ΔLVEF, Δ6MWT, ΔMLFHQ) in persistent AF patients are reported in the Supplementary Materials, Figure S5.

## 4. Discussion

Herein, we performed a systematic review and meta-analysis to provide a comprehensive overview of the outcomes of HF patients undergoing CA versus MT of AF.

We observed that CA was associated with a 35% reduction in the composite endpoint (all-cause mortality and HF hospitalization) compared to MT. A significant reduction was observed when the endpoints above were assessed separately (all-cause mortality: −35%; HF hospitalization: −33%).

Regarding other secondary outcomes, CA led to a lower rate of AF recurrence and CV death and promoted a significant improvement in the left ventricular systolic function (ΔLVEF), exercise capacity (Δ6MWT), cardiorespiratory fitness (ΔVO_2_ max), quality of life (ΔMLHFQ), and HF severity (ΔBNP).

To the best of our knowledge, our systematic review and meta-analysis is the most updated and comprehensive analysis of RCTs thus far. Compared to prior studies [28,29], we included data from the two recent AMICA [13] and RAFT-AF [14] trials, which together account for approximately one third of our study population, and performed additional endpoint/subpopulation analyses.

CA has been demonstrated to be the most effective rhythm control strategy, with several recent trials showing its superiority compared to MT as an early AF therapy in the general population [30]. Nonetheless, it is far more challenging to achieve rhythm control in patients with a high burden of comorbidities (e.g., HF, obstructive sleep apnea, coronary artery disease) [3,26,31,32,33] due to their more diseased atrial substrate. These patients are also more prone to drug-related, as well as ablation-related, complications.

AF and HF often coexist and share several risk factors, which may contribute to arrhythmia progression, as well as worse clinical outcomes, including stroke, hospitalization, and overall and cardiovascular mortality. RCTs investigating the success rate of CA compared to MT in patients with AF and HF have provided conflicting results. Among them, data from the two recent RCTs, AMICA [13] and RAFT [14], were published after the enrolment was closed early due to their apparent futility [13,14].

The RAFT-AF [14] trial enrolled 411 patients with high-burden paroxysmal or persistent AF. Although the initial goal was to recruit 600 patients, a determination of apparent futility by the Data Safety Monitoring Committee after an interim analysis led to the trial’s early termination. A non-significant trend towards improved outcomes with CA was reported, with the HR value of 0.71 (95% CI: 0.49–1.03; *p* = 0.066). Notably, CA led to a significantly greater improvement in LVEF, exercise capacity, AF and HF symptoms, and quality of life.

The AMICA [13] trial enrolled patients with persistent/longstanding persistent AF and LVEF ≤35%, aiming to assess any LVEF changes after 1 year. The study revealed a similar improvement of the LVEF with CA and MT; however, the comparison between the two treatment arms showed no statistically significant difference. The only significant difference was observed in the device-recorded AF burden, which was significantly higher in the MT group. It can be speculated that the trial included patients with a higher comorbidity rate compared to other similar trials (e.g., CASTLE-AF) [18], who were potentially too sick to show any significant clinical and functional advantages from CA and the resulting better sinus rhythm control. Similar findings were reported in the subpopulation of CASTLE-AF patients with New York Heart Association (NYHA) functional class III symptoms or an LVEF of <25%, who did not show any significant benefits from CA. In the CAMERA-MRI [24] trial, the improvement in LVEF promoted by CA was significantly better, with systolic function normalization being achieved in 58% of patients compared to 9% of those on MT (*p* = 0.0002). Nonetheless, the absolute LVEF improvement was significantly greater among patients without late gadolinium enhancement [3]. Thus, advanced cardiac remodeling may limit the recovery of the LVEF and also increase the risk of AF relapse in the long term [26,32]. These observations highlight the importance of an early AF ablation strategy.

CA, as a first line approach, might be particularly important for HF patients, since arrhythmic recurrences promote worsening cardiomyopathy, arrhythmia progression, and poor outcomes. Once the vicious cycle of AF and HF begins, the success rate of PVI is significantly lower and other sources of triggers outside the PVs may contribute to arrhythmia initiation/relapse [4]. Therefore, early ablation is critical for HF patients, as it is associated with better ablation success. Mechanistic and clinical studies have also highlighted the dynamic interplay between sinus rhythm restoration, cardiac function, symptoms, and clinical outcomes. Specifically, successful rhythm control may subsequently promote positive atrial and ventricular remodeling, which may result in better clinical outcomes. From this perspective, the benefits of CA on the cardiac function may manifest themselves later, and outcome improvements may occur even later. From a functional standpoint, the PABA-CHF [34] trial compared PVI versus atrioventricular-node ablation combined with biventricular pacing in HF patients with EF < 40%. Patients were followed-up over 6 months, showing a steady improvement in the mean LVEF in the CA group, who continued to recover until the end of the study period. From a clinical standpoint, the mortality curves separated only after 2 years in the CASTLE-AF [18] trial. Further studies are necessary in order to understand the best AF ablation strategy for HF patients, as well as the pathophysiological basis of CA-mediated functional and clinical benefits.

Another finding worth highlighting is the safety profile of CA in a population such as the HF population, characterized by a high comorbid profile. Notably, CA appeared to be safe and showed a similar risk of adverse events as MT. The prevalence of procedural complications was 5.02% (95% CI: 3.44–6.81), with access-site complications accounting for approximately half of the events [28,35,36].

## 5. Limitations

Our study has several limitations that need to be acknowledged. (1) Patients selected for a randomized catheter ablation trial may be healthier than those in real-world situations. (2) The number of patients enrolled in each RCTs was highly variable and may account for the imbalance in the results and heterogeneity. (3) The high heterogeneity regarding the arrhythmia detection techniques during follow-up should be considered. (4) Additional ablation outside the PVs, performed in some RCTs, could have affected the clinical outcomes. (5) The RCTs included here enrolled patients from 2011 to 2022, involving temporal changes in both CA and drug therapy. (6) Because patients and physicians were not blinded to the treatment assignment, it is possible that the post-ablation medical management differed between RCTs.

## 6. Conclusions

AF ablation was superior to conventional drug therapy in improving the composite of all-cause mortality and HF hospitalization, the all-cause mortality, HF hospitalization, AF recurrence, cardiovascular death, LVEF, 6 min walk test distance, VO_2_ max, and quality of life.

## Figures and Tables

**Table 1:** Study Baseline Characteristics of Patients Included in the Analysis.

Study		MacDonald et al., 2011 [23]	ARC-HF, 2013 [21]	CAMTAF, 2014 [20]	AATAC, 2016 [19]	CAMERA-MRI, 2017 [24]	CASTLE-AF, 2018 [18]	AMICA, 2019 [13]	CAMERA LATE OUTCOMES, 2020 [22]	CABANA, 2021 [17]	RAFT-AF, 2022 [14]
**Monocentric or multicentric**		Multicentric	Multicentric	Monocentric	Multicentric	Multicentric	Multicentric	Multicentric	Multicentric	Multicentric	Multicentric
**Comparison**		Ablation vs. medical rate control	Ablation vs. medical rate control	Ablation vs. medical rate control	Ablation vs. amiodarone	Ablation vs. medical rate control	Ablation vs. medical rhythm and rate control	Ablation vs. medical rhythm and rate control	Ablation vs. medical rate control	Ablation vs. medical rhythm and rate control	Ablation vs. medical rate control
**HF inclusion criteria**		NYHA class II or greater and optimal HF treatment for at least 3 months	NYHA class II or greater and optimal HF treatment for at least 1 month	NYHA class II or greater and optimal HF treatment for at least 3 months	NYHA class II to III	NYHA class ≥ II	NYHA class ≥ II	NYHA class II or greater and optimal HF treatment for at least 1 months	NYHA class ≥ II	NYHA class ≥ II	NYHA class II/III HF on optimal guideline directedmedical therapy and elevated NT-proBNP
**LVEF inclusion criterion**		≤35% (RNVG)	≤35%	<50%	<40%	≤45%	≤35%	≤35%	≤45%	No LVEF inclusion criterion	No LVEF inclusion criterion
**Type of AF**		Persistent	Persistent	Persistent	Persistent	Persistent	Paroxysmal or persistent	Persistent	Persistent	Paroxysmal or persistent	Paroxysmal or persistent
**Patients at randomization, n**	Ablation	22	26	26	102	34	200	104	34	378	214
	Drug	19	26	24	101	34	197	98	34	400	197
**Mean age, years (SD or IQR)**	Ablation	62.3 ± 6.7	64 ± 10	55 ± 12	62 ± 10	59 ± 11	64 (56–71)	65 ± 8	60.5 ± 10.7	68 (62, 73)	65.9 ± 8.6
	Drug	64.4 ± 8.3	62 ± 9	60 ± 10	60 ± 11	62 ± 9.4	64 (56–73.5)	65 ± 8	65.5 ± 7.2	67 (62, 73)	67.5 ± 8.0
**LVEF Baseline (SD or IQR), %**	Ablation	36.1 ± 11.9 (MRI) 16.1 ± 7.1 (RNVG)	22 ± 8 (RNVG)	31.8 ± 7.7	29 ± 5	35 ± 9.8 (MRI)	32.5 (25.0–38.0)	27.8 ± 9.5	36.1 ± 9.6 (MRI)	55 (50-60)	EF ≤ 45%: 30.1 ± 8.5 EF > 45%: 55.9 ± 6.7
	Drug	42.9 ± 9.6 (MRI) 19.6 ± 5.5 (RNVG)	25 ± 7 (RNVG)	33.7 ± 12.1	30 ± 8	35 ± 9.3 (MRI)	31.5 (27.0–37.0)	24.8 ± 8	34.6 ± 9.1 (MRI)	56 (50-62)	EF ≤ 45%: 30.3 ± 9.2 EF > 45%: 54.6 ± 7.3
**Mean baseline 6MWT (SD), meters**	Ablation	317.5 ± 125.8	416 ± 78	NA	348 ± 111	491 ± 147	NA	NA	NA	NA	363.1 ± 101.4
	Drug	351.8 ± 117.1	411 ± 109	NA	350 ± 130	489 ± 132	NA	NA	NA	NA	344.4 ± 107.1
**Mean baseline VO_2_ max (SD), mL/kg per min**	Ablation	NA	16.3 ± 5.3	22	NA	NA	NA	NA	NA	NA	NA
	Drug	NA	18.2 ± 4.8	19.5	NA	NA	NA	NA	NA	NA	NA
**Mean baseline MLHFQ score (SD)**	Ablation	55.8 ± 19.8	42 ± 23	42	52 ± 24	NA	NA	NA	NA	NA	NA
	Drug	59.2 ± 22.4	49 ± 21	48	50 ± 27	NA	NA	NA	NA	NA	NA
**Mean baseline BNP(SD or IQR), pg/mL**	Ablation	NA	412 ± 324	NA	NA	266 ± 210	NA	NA	NA	NA	NA
	Drug	NA	283 ± 285	NA	NA	256 ± 208	NA	NA	NA	NA	NA
**Follow-up**		6 mo	12 mo	6 and 12 mo	24 mo	6 mo	60 mo	12 mo	4.0 ± 0.9 years	48.5 mo	24 mo

Note: 6MWT: 6 min walk test; AATAC: ablation versus amiodarone for treatment of atrial fibrillation in patients with congestive heart failure and an implanted ICD; AF: atrial fibrillation; AMICA: atrial fibrillation management in congestive heart failure with ablation; ARC-HF: a randomized trial to assess catheter ablation versus rate control in the management of persistent atrial fibrillation in chronic heart failure; BNP: brain natriuretic peptide; CABANA: catheter ablation vs. antiarrhythmic drug therapy for atrial fibrillation; CAMERA-MRI: catheter ablation versus medical rate control in atrial fibrillation and systolic dysfunction; CAMTAF: a randomized controlled trial of catheter ablation versus medical treatment of atrial fibrillation in heart failure; CASTLE-AF: catheter ablation versus standard conventional therapy in patients with left ventricular dysfunction and atrial fibrillation; HF: heart failure; IQR: interquartile range; LVEF: left ventricular ejection fraction; MLHFQ: Minnesota Living with Heart Failure Questionnaires; mo: months; MRI: magnetic resonance imaging; NA: not available; NYHA: New York Heart Association; RAFT: randomized ablation-Based rhythm control versus rate control; RNVG: radionuclide ventriculography; SD: standard deviation; VO_2_ max: peak oxygen consumption.

**Table 2:** Periprocedural Complications of Catheter Ablation (A) and Adverse Events of Antiarrhythmic Drugs (B).

A. Periprocedural Complications								
Study	Access Site Complications, n	Pericardial Effusion/tamponade, n		Esophageal Complications, n		Systemic Embolism, n		Pulmonary Stenosis, n
MacDonald et al., 2011 [23]	0	2		0		0		0
ARC-HF, 2013 [21]	1	1		0		0		0
CAMTAF, 2014 [20]	0	1		0		1		0
AATAC, 2016 [19]	2	1		0		0		0
CAMERA-MRI, 2017 [24]	1	0		0		0		0
CASTLE-AF, 2018 [18]	3	3		0		0		1
AMICA, 2019 [13]	2	1		1		0		0
CABANA, 2021 [17]	15	2		4		0		0
RAFT-AF, 2022 [14]	9	6		1		4		0
OVERALL, %	2.37%	0.8%		0.07%		0.01%		0.001%
**B. Antiarrhythmic Drug Adverse Events**								
**Study**	**Thyroid toxicity, n**		**Liver and Pulmonary toxicity, n**		**Proarrhythmic effect, n**		**Unspecified toxicity, n**	
AATAC, 2016 [19]	4		3					
CABANA, 2021 [17]	9		2		3		5	
RAFT-AF, 2022 [14]					4		1	
OVERALL, %	1.38%		0.48%		0.8%		0.7%	

## Data Availability

Data available in a publicly accessible repository.

## Supplementary Materials

The following supporting information can be downloaded at: https://www.mdpi.com/article/10.3390/jcm11195530/s1, Expanded Methods; Full Search Strategy and Search Terms; Data Extraction; Expanded Results; Table S1: Additional Baseline Clinical Characteristics. Table S2: Overview of Ablation Strategies in Each Randomized Controlled Trial. Table S3: Thromboembolic Events during Follow-Up. Figure S1: Methodological evaluation of the included studies. Figure S2: Any Adverse Events and Thromboembolic Events. Figure S3: Catheter Ablation vs Medical Rate Control. Figure S4: Catheter Ablation vs Drug Therapy in patients with LVEF ≤ 50%. Figure S5: Catheter Ablation vs Drug Therapy in patients with Persistent AF.

## Abbreviations

AATAC	Ablation versus Amiodarone for Treatment of Atrial Fibrillation in Patients with Congestive Heart Failure and an Implanted ICD
AF	Atrial Fibrillation
AMICA	Atrial Fibrillation Management in Congestive Heart Failure with Ablation
ARC-HF	A Randomized Trial to Assess Catheter Ablation Versus Rate Control in the Management of Persistent Atrial Fibrillation in Chronic Heart Failure
BNP	Brain Natriuretic Peptide
CA	Catheter Ablation
CABANA	Catheter Ablation vs. Antiarrhythmic Drug Therapy for Atrial Fibrillation
CAD	Coronary Artery Disease
CAMERA-MRI	Catheter Ablation Versus Medical Rate Control in Atrial Fibrillation and Systolic Dysfunction
CAMTAF	A Randomized Controlled Trial of Catheter Ablation Versus Medical Treatment of Atrial Fibrillation in Heart Failure
CASTLE-AF	Catheter Ablation versus Standard Conventional Therapy in Patients with Left Ventricular Dysfunction and Atrial Fibrillation
CIED	Cardiac Implantable Electronic Device
CRT-D	Cardiac Resynchronization Therapy Defibrillator
CV	Cardiovascular
ECG	Electrocardiogram
HF	Heart Failure
LVEF	Left Ventricular Ejection Fraction
MLHFQ	Minnesota Living with Heart Failure Questionnaire
MT	Medical Therapy
MRI	Magnetic Resonance Imaging
NYHA	New York Heart Association
PRISMA	Preferred Reporting Items for Systematic Reviews and Meta-Analyses
PVI	Pulmonary Vein Isolation
RAFT	Randomized Ablation-Based Rhythm-Control Versus Rate-Control
RCT	Randomized Controlled Trial
RR	Risk Ratio
SVC	Superior Vena Cava
VO_2_ max	Peak Oxygen Consumption
6MWT	6-Min Walk Test
