# Supplementary material for: Catheter Ablation versus Medical Therapy of Atrial Fibrillation in Patients with Heart Failure: An Updated Systematic Review and Meta-Analysis of Randomized Controlled Trials
Source: J Clin Med. 2022 Sep 21;11(19):5530. doi: 10.3390/jcm11195530 (PMC9572511; doi:10.3390/jcm11195530)
Supplement: Supplementary file 1 [file jcm-11-05530-s001.zip › jcm-1904980-supplementary.pdf]

SUPPLEMENTAL MATERIALS

Catheter Ablation Versus Medical Therapy of Atrial Fibrillation in Patients with Heart Failure: A Systematic Review and Meta-Analysis of Randomized Controlled Trials

Expanded Methods

Full Search Strategy and Search Terms

Data Extraction

Expanded Results

Overview of Trials

Supplemental Tables

Supplemental Table S1

Supplemental Table S2

Supplemental Table S3

Supplemental Figures

Supplemental Figure S1

Supplemental Figure S2

Supplemental Figure S3

Supplemental Figure S4

Supplemental Figure S5

Expanded Methods

Full Search Strategy and Search Terms.

Three investigators (A.P, G.V and M.M.) conducted a literature search in searched Medline, Cochrane, Journals@Ovid, Scopus electronic databases for RCTs published from inception to 26th May 2022. Preferred Reporting Items for Systematic Reviews and Meta-Analyses (PRISMA) guidelines were adopted. PubMed search terms were (“atrial fibrillation”) AND (“heart failure”); (“atrial fibrillation”) AND (“left ventricular dysfunction”); (“atrial fibrillation”) AND (“catheter ablation”); (“catheter ablation”) AND (“left ventricular dysfunction”); (“heart failure”) AND (“catheter ablation”); (“drug therapy”) AND (“left ventricular dysfunction”); (“heart failure”) AND (“radiofrequency ablation”); (“atrial fibrillation”) AND (“radiofrequency ablation”); (“drug therapy”) AND (“atrial fibrillation”); (“drug therapy”) AND (“radiofrequency ablation”) AND (“heart failure”); (“drug therapy”) AND (“radiofrequency ablation”) AND (“atrial fibrillation”); (“drug therapy”) AND (“radiofrequency ablation”) AND (“left ventricular dysfunction”); (“atrial fibrillation”) AND (“radiofrequency ablation”) AND (“heart failure”); (“atrial fibrillation”) AND (“radiofrequency ablation”) AND (“left ventricular dysfunction”); (“atrial fibrillation”) AND (“drug therapy”) AND (“heart failure”). Embase search terms were (“catheter ablation” OR “drug therapy”) AND (“heart failure” OR “atrial fibrillation”). Details of the search algorithm are shown in Online Figure 1.

Data Extraction

Among the RCTs included, data were extracted from: 1) a sub-analysis of the Catheter Ablation vs Antiarrhythmic Drug Therapy for Atrial Fibrillation (CABANA) trial including HF patients with NYHA class ≥ II [17]; 2) the long-term outcomes of the Catheter Ablation Versus Medical Rate Control in Atrial Fibrillation and Systolic Dysfunction (CAMERA-MRI) study, with the exception of Δ6MWT and ΔBNP (evaluated at 6 months), since they were not reported in the 5year follow-up

study; the meta-analysis by Al Halabi et al. [25], because some outcomes of MacDonald et al. and A Randomized Trial to Assess Catheter Ablation Versus Rate Control in the Management of Persistent Atrial Fibrillation in Chronic Heart Failure (ARC-HF) trials were not present in the original studies (the two first authors of the trials are co-authors of the meta-analysis).

Expanded Results

Overview of Trials

Six RCTs included patients with persistent AF with a mean duration >12 month with the exception of the Ablation versus Amiodarone for Treatment of Atrial Fibrillation in Patients with Congestive Heart Failure and an Implanted ICD (AATAC), which included patients with an average AF duration of 8.2 ± 3.6 months. Three other RCTs included also patients with paroxysmal AF [56% CASTLE-AF (Catheter Ablation versus Standard Conventional Therapy in Patients with Left Ventricular Dysfunction and Atrial Fibrillation); 7.2% RAFT-AF (Randomized Ablation-Based Rhythm-Control Versus Rate-Control); 31.6% CABANA]. Seven trials enrolled HF patients with NYHA class II-III; CAMERA-MRI and CASTLE-AF also included a small percentage of patients (1.3%) with NYHA class IV. Regarding left ventricular systolic function, 79% patients in CABANA trial had a LVEF ≥50%, 11.7% had a LVEF between 40% and 49%, and 9.3% had an LVEF <40%. In RAFT-AF trial, 57.9% had a LVEF≤45 and 42.1% had a LVEF>45%. In CAMERA-MRI, 49% patients had LVEF<35%. CAMTAF (A Randomized Controlled Trial of Catheter Ablation Versus Medical Treatment of Atrial Fibrillation in Heart Failure) trial had a selection criterion of LVEF<50% but did not perform a sub-analysis according to LVEF class.

Supplemental Tables

Supplemental Table S1. Additional Baseline Clinical Characteristics.

Study		MacDonald et al, 2011[23]	ARC-HF, 2013[21]	CAMTAF, 2014[20]	AATAC, 2016[19]	CAMERA-MRI, 2017[24]	CASTLE-AF, 2018[18]	AMICA, 2019[13]	CAMERA LATE OUTCOMES, 2020[22]	CABANA, 2021[17]	RAFT-AF, 2022[14]
**Patients, n**		41	52	50	203	66	363	195	66	778	411
**Male sex, n (%)**	Ablation	17 (77)	21 (81)	25	77 (75)	31 (94)	156 (87)	60 (88)	42 (89.3)	207 (54.8)	157 (73.4)
	Drug Therapy	15 (79)	24 (92)	23	74 (73)	29 (88)	155 (84)	66 (92)	14 (93.3)	226 (56.5)	148 (75.1)
**Period of pharmacologic optimization, month (mo)**		3 mo	1 mo	3 mo	3 mo	1 mo	5 mo	3 mo	1 mo		1.5 mo
**β-Blockers, n (%)**	Ablation	18 (82)	24 (92)	NA	78 (76)	32 (97)	165 (93)	62 (91)	47 (100.0)	284 (75)	197 (92.1)
	Drug Therapy	18 (95)	24 (92)	NA	81 (80)	32 (97)	174 (95)	67 (93)	14 (93.3)	300 (75)	182 (92.4)
**ACEI/ARB, n (%)**	Ablation	21 (95)	25 (96)	NA	94 (92)	31 (94)	166 (94)	62 (91)	45 (95.7)	NA	155 (72.4)
	Drug Therapy	18 (95)	26 (100)	NA	89 (88)	31 (94)	164 (91)	68 (94)	14 (93.3)	NA	161 (81.7)
**Digoxin, n (%)**	Ablation	12 (55)	16 (62)	NA	NA	NA	31 (18)	20 (29)	NA	49 (13)	55 (25.7)
	Drug Therapy	9 (47)	12 (46)	NA	NA	NA	56 (31)	21 (29)	NA	48 (12)	65 (33.0)
**Aldosterone antagonist, n (%)**	Ablation	10 (45)	13 (50)	NA	46 (45)	11 (33)	NA	44 (65)	17 (36.2)	NA	51 (23.8)
	Drug Therapy	3 (16)	6 (23)	NA	51 (50)	16 (48)	NA	48 (67)	5 (33.3)	NA	53 (26.9)
**Antiarrhythmics, n (%)**	Ablation	NA	NA	NA	NA	8 (24)	57 (32%)	17 (25)	14 (29.8)	94 (25)	NA
	Drug Therapy	NA	NA	NA	NA	8 (24)	55 (31%)	27 (38)	2 (13.3)	116 (29)	NA
**Type of antiarrhythmics**		NA	NA	NA	Amiodarone	NA	class Ia, Ic, or III	Amiodarone	NA	class Ia, Ic, or III	NA
**Mean time in AF (SD or IQR), mo**	Ablation	44±36.5	51±39	24 (17–33)	8.6±3.2	23±18	NA	NA	2.4±2.5 years	1.1 (0.2-3.7) years	14.5 (7-36)
	Drug Therapy	64±47.6	51±76	24 (12–48)	8.4±4.1	21±15	NA	NA	2.0±1.7 years	1.2 (0.3-4.2) years	15 (6-48)
**NYHA Class**		II and III	II and III	II and III	II and III	II, III, and IV	I, II, III, and IV	II and III	II, III, and IV	II and III	II and III
**ICD/CRTD, n**		NA	14	NA	203	NA	363	192	5	NA	135
**CAD, n (%)**	Ablation	11 (50)	10 (38)	6	63 (62)	0	72 (40)	30 (44)	NA	80 (21.2%)	74 (34.6)
	Drug Therapy	9 (47)	7 (27)	7	66 (65)	0	96 (52)	40 (56)	NA	90 (22.5%)	55 (27.9)
**LA-Diameter (SD or IQR), mm**	Ablation	NA	50±6	5.2±1.1	47±4.2	48±5.5	48.0 (45.0–54.0)	50±6	NA	NA	46.1±6.0
	Drug Therapy	NA	46±7	5.0±1.0	48±4.9	47±8.2	49.5 (5.0–55.0)	51±5	NA	NA	46.8±5.4
**Frequency of monitoring, mo**		3 and 6	3, 6 and 12	1, 3, and 6	3, 6, 12 and 24	6	3, 6, 12, 24, 36, 48, and 60	1, 3, 6, 12	6,12,18,24,30,36,42 ,48	6,12,18,24,30,36,42 ,48,54,60	2, 4, 6, 12, 18 and 24
**Monitoring method**		24-h Holter	48-h Holter, CIED	48-h Holter	CIED	ILR	CIED	CIED	CIED or 24-h Holter	96-h Holter	14 day continuos ECG

AATAC: *Ablation versus Amiodarone for Treatment of Atrial Fibrillation in Patients with Congestive Heart Failure and an Implanted ICD;* ***ACEI:*** *Angiotensin-Converting Enzyme Inhibitor;* ***AF:*** *Atrial Fibrillation;* ***AMICA:*** *Atrial Fibrillation Management in Congestive Heart Failure With Ablation;* ***ARB:*** *Angiotensin-Receptor Blocker;* ***ARC-HF:*** *A Randomized Trial to Assess Catheter Ablation Versus Rate Control in the Management of Persistent Atrial Fibrillation in Chronic Heart Failure;* ***CABANA:*** *Catheter Ablation vs Antiarrhythmic Drug Therapy for Atrial*

Fibrillation; ***CAMERA-MRI:*** *Catheter Ablation Versus Medical Rate Control in Atrial Fibrillation and Systolic Dysfunction;* ***CAMTAF:*** *A Randomized Controlled Trial of Catheter Ablation Versus Medical Treatment of Atrial Fibrillation in Heart Failure;* ***CASTLE-AF:*** *Catheter Ablation versus Standard Conventional Therapy in Patients with Left Ventricular Dysfunction and Atrial Fibrillation;* ***CAD:*** *Coronary Artery Disease;* ***CIED****: Cardiac Implantable Electronic Device;* ***CRT-D:*** *Cardiac Resynchronization Therapy Defibrillator;* ***HF:*** *Heart Failure;* ***ICD:*** *Implantable Cardioverter-Defibrillator;* ***ILR:*** *Implantable Loop Recorder;* ***IQR:*** *Interquartile range;* ***LA:*** *Left Atrium;* ***LVEF:*** *Left Ventricular Ejection Fraction;* ***mo****: Months;* ***NA:*** *Not Available;* ***NYHA:*** *New York Heart Association;* ***PVI:*** *Pulmonary Vein Isolation;* ***RAFT:*** *Randomized Ablation-Based Rhythm-Control Versus Rate-Control;* ***SD:*** *Standard Deviation.*

Supplemental Table S2. Overview of Ablation Strategies in Each Randomized Controlled Trial.

Study	PVI	CFAE Ablation	Posterior Wall Isolation	SVC Isolation	Elimination of AF Triggers	Linear Ablation of the Left Atrial Roof, Cavotricuspid Isthmus and/or Mitral Isthmus
MacDonald et al, 2011 [23]	×	×				×
ARC-HF, 2013 [21]	×	×				×
CAMTAF, 2014 [20]	×	×				×
AATAC, 2016 [19]	×	×	×	×	×	×
CAMERA-MRI, 2017 [24]	×		×			
CASTLE-AF, 2018 [18]	×	×	×	×	×	×
AMICA, 2019 [13]	×	×				×
CABANA, 2021 [17]	×	×	×	×	×	
RAFT-AF, 2022 [14]	×	×	×		×	

AATAC: *Ablation versus Amiodarone for Treatment of Atrial Fibrillation in Patients with Congestive Heart Failure and an Implanted ICD;* ***AMICA:*** *Atrial Fibrillation Management in Congestive Heart Failure With Ablation;* ***AF:*** *Atrial Fibrillation;* ***ARC-HF:*** *A Randomized Trial to Assess Catheter Ablation Versus Rate Control in the Management of Persistent Atrial Fibrillation in Chronic Heart Failure;* ***CABANA:*** *Catheter Ablation vs Antiarrhythmic Drug Therapy for Atrial Fibrillation;* ***CFAE:*** *Complex Fractionated Atrial Electrograms;* ***CAMERA-MRI:*** *Catheter Ablation Versus Medical Rate Control in Atrial Fibrillation and Systolic Dysfunction;* ***CAMTAF:*** *A Randomized Controlled Trial of Catheter Ablation Versus Medical Treatment of Atrial Fibrillation in Heart Failure;* ***CASTLE-AF:*** *Catheter Ablation versus Standard Conventional Therapy in Patients with Left Ventricular Dysfunction and Atrial Fibrillation;* ***PVI:*** *Pulmonary Vein Isolation;* ***RAFT:*** *Randomized Ablation-Based Rhythm-Control Versus Rate-Control;* ***SVC:*** *Superior Vena Cava.*

Supplemental Table S3. Thromboembolic Events during Follow-Up.

Study	Ischaemic stroke, n		Haemorrhagic stroke, n	
	Ablation	Drug Therapy	Ablation	Drug Therapy
**MacDonald et al, 2011 [23]**	1	0	0	0
**ARC-HF, 2013 [21]**	0	1	0	0
**CAMTAF, 2014 [20]**	0	0	0	1
**CAMERA-MRI, 2017 [24]**	0	0	0	0
**CASTLE-AF, 2018 [18]**	5	11	0	0
**RAFT-AF, 2022 [14]**	1	5	0	0
**OVERALL, %**	**2.60%**		**0.48%**	

ARC-HF: *A Randomized Trial to Assess Catheter Ablation Versus Rate Control in the Management of Persistent Atrial Fibrillation in Chronic Heart Failure;* ***CAMERA-MRI:*** *Catheter Ablation Versus Medical Rate Control in Atrial Fibrillation and Systolic Dysfunction;* ***CAMTAF:*** *A Randomized Controlled Trial of Catheter Ablation Versus Medical Treatment of Atrial Fibrillation in Heart Failure;* ***CASTLE-AF:*** *Catheter Ablation versus Standard Conventional Therapy in Patients with Left Ventricular Dysfunction and Atrial Fibrillation;* ***RAFT:*** *Randomized Ablation-Based Rhythm-Control Versus Rate-Control.*

Supplemental Figures

Supplemental Figure S1. Methodological evaluation of the included studies. (A) Methodological quality summary and (B) methodological quality graph for the risk of bias from the included randomized controlled trials using the six domains of the Cochrane tool.

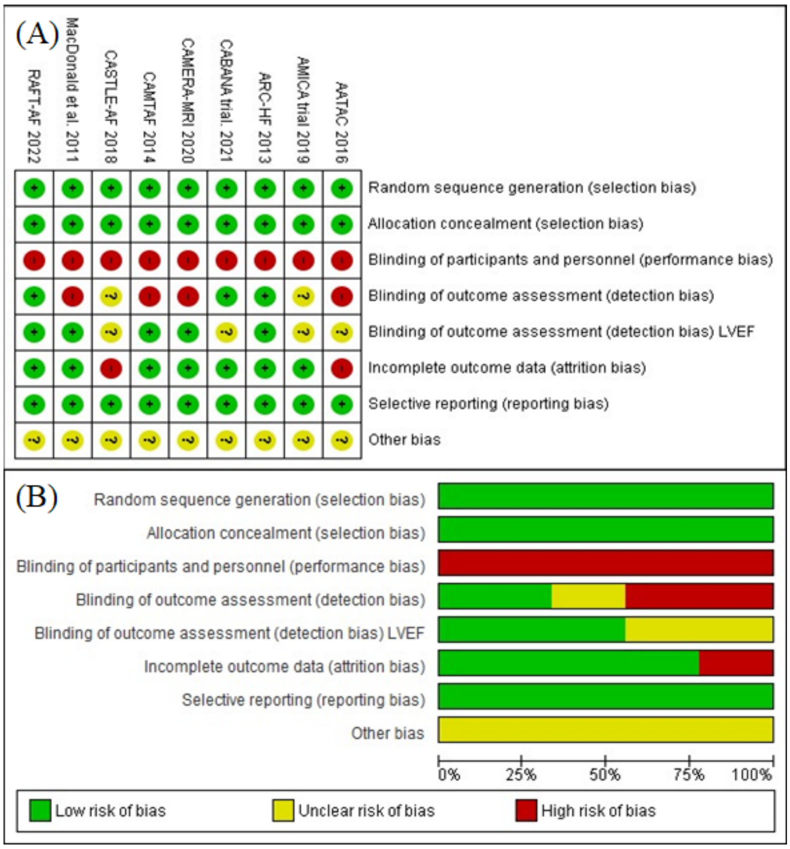

***AATAC:** Ablation versus Amiodarone for Treatment of Atrial Fibrillation in Patients with Congestive Heart Failure and an Implanted ICD; **AMICA:** Atrial Fibrillation Management in Congestive Heart Failure With Ablation; **ARC-HF:** A Randomized Trial to Assess Catheter Ablation Versus Rate Control in the Management of Persistent Atrial Fibrillation in Chronic Heart Failure; **CABANA:** Catheter Ablation vs Antiarrhythmic Drug Therapy for Atrial Fibrillation; **CAMERA-MRI:** Catheter Ablation Versus Medical Rate Control in Atrial Fibrillation and Systolic Dysfunction**; CAMTAF:** A Randomized Controlled Trial of Catheter Ablation Versus Medical Treatment of Atrial Fibrillation in Heart Failure; **CASTLE-AF:** Catheter Ablation versus Standard Conventional Therapy in Patients with Left Ventricular Dysfunction and Atrial Fibrillation; **LVEF:** Left Ventricular Ejection Fraction; **RAFT:** Randomized Ablation-Based Rhythm-Control Versus Rate-Control.*

Supplemental Figure S2. Any Adverse Events and Thromboembolic Events. Forest plots displaying the incidence of Adverse Events (a) and Thromboembolic Events (b) in patients with AF and HF undergoing AF ablation strategy versus drug therapy strategy.

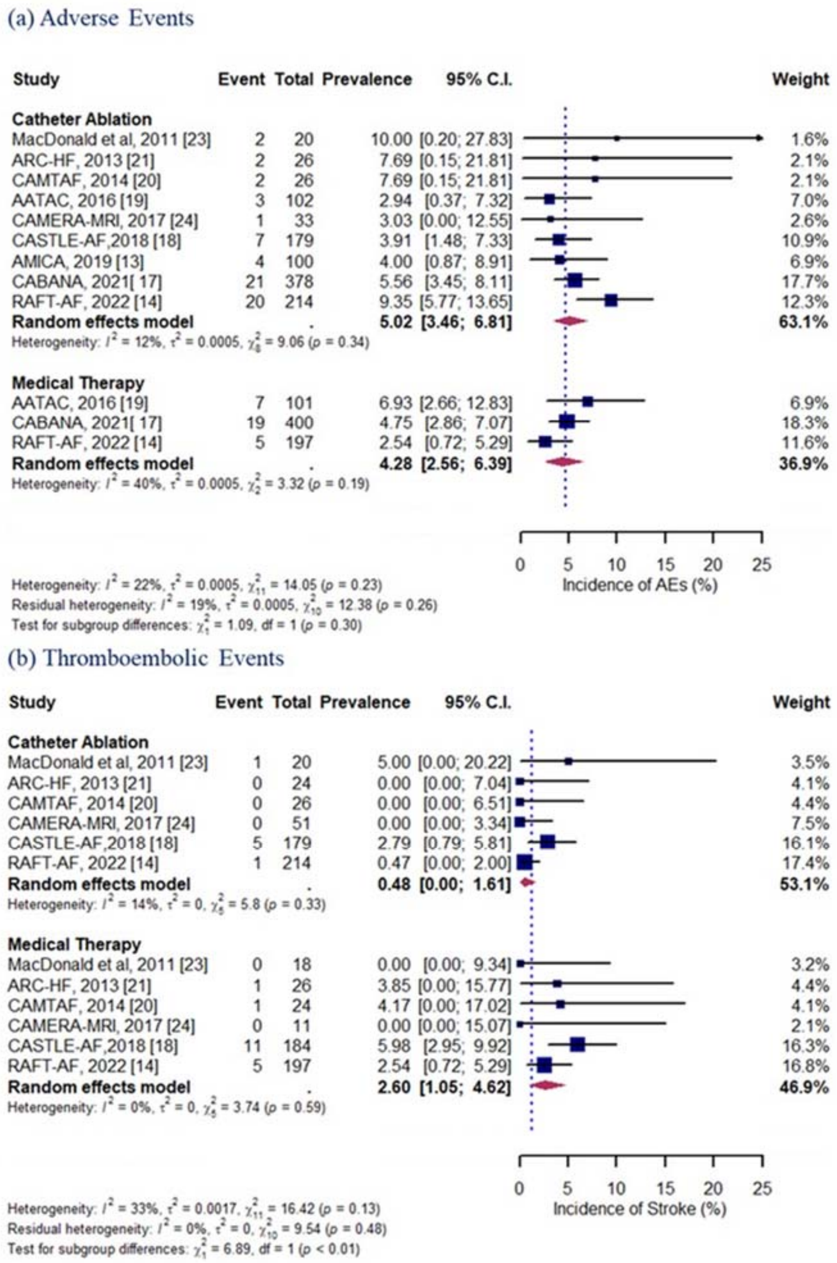

***AATAC:** Ablation versus Amiodarone for Treatment of Atrial Fibrillation in Patients with Congestive Heart Failure and an Implanted ICD; **AEs:** Adverse Events; **AMICA:** Atrial Fibrillation Management in Congestive Heart Failure With Ablation; **ARC-HF:** A Randomized Trial to Assess Catheter Ablation Versus Rate Control in the Management of Persistent Atrial Fibrillation in Chronic Heart Failure; **CABANA:** Catheter Ablation vs Antiarrhythmic Drug Therapy for Atrial Fibrillation; **CAMERA-MRI:** Catheter Ablation Versus Medical Rate Control in Atrial Fibrillation and Systolic Dysfunction; **CAMTAF:** A Randomized Controlled Trial of Catheter Ablation Versus Medical Treatment of Atrial Fibrillation in Heart Failure; **CASTLE-AF:** Catheter Ablation versus Standard Conventional Therapy in*

Patients with Left Ventricular Dysfunction and Atrial Fibrillation; ***CI:*** *Confidence Interval;* ***RAFT:*** *Randomized Ablation-Based Rhythm-Control Versus Rate-Control.*

Supplemental Figure S3. Catheter Ablation vs Medical Rate Control. Forest plots displaying mean differences in all-cause mortality (a), HF hospitalization (b), CV death (c), AF recurrence (d), LVEF (e), 6MWT (f) and MLFHQ (g) between ablation and medical rate control.

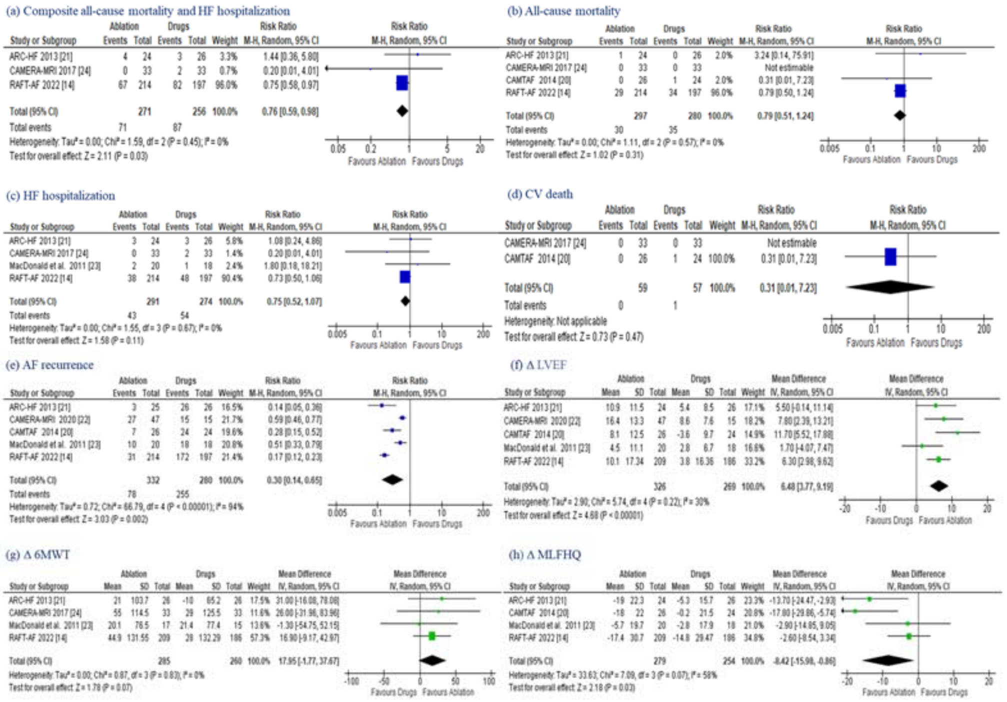

***6MWT:** 6 Minute Walk Test; **AF:** Atrial Fibrillation; **ARC-HF:** A Randomized Trial to Assess Catheter Ablation Versus Rate Control in the Management of Persistent Atrial Fibrillation in Chronic Heart Failure; **CAMERA-MRI:** Catheter Ablation Versus Medical Rate Control in Atrial Fibrillation and Systolic Dysfunction; **CAMTAF:** A Randomized Controlled Trial of Catheter Ablation Versus Medical Treatment of Atrial Fibrillation in Heart Failure; **CI:** Confidence Interval; **CV:** Cardiovascular; **HF:** Heart Failure; **LVEF:** Left Ventricular Ejection Fraction; **MLHFQ:** Minnesota Living with Heart Failure Questionnaires; **SD:** Standard Deviation; **RAFT:** Randomized Ablation-Based Rhythm-Control Versus Rate-Control.*

Supplemental Figure S4. Catheter Ablation vs Drug Therapy in patients with LVEF ≤ 50%. Forest plots displaying mean differences in all-cause mortality (a), HF hospitalization (b), CV death (c), AF recurrence (d), LVEF (e), 6MWT (f) and MLFHQ (g) between ablation and drug therapy in patients with LVEF ≤ 50%.

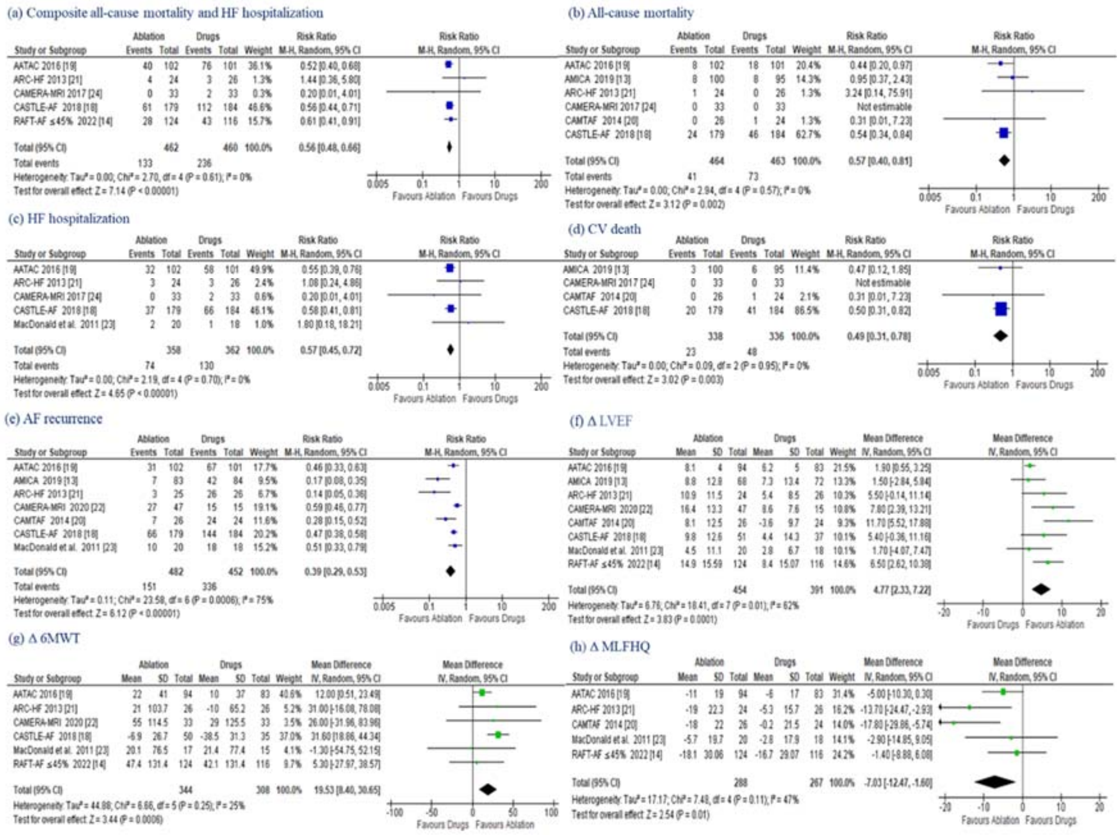

***6MWT:** 6 Minute Walk Test; **AF:** Atrial Fibrillation; **AATAC:** Ablation versus Amiodarone for Treatment of Atrial Fibrillation in Patients with Congestive Heart Failure and an Implanted ICD; **AMICA:** Atrial Fibrillation Management in Congestive Heart Failure With Ablation; **ARC-HF:** A Randomized Trial to Assess Catheter Ablation Versus Rate Control in the Management of Persistent Atrial Fibrillation in Chronic Heart Failure**; CAMERA-MRI:** Catheter Ablation Versus Medical Rate Control in Atrial Fibrillation and Systolic Dysfunction; **CAMTAF:** A Randomized Controlled Trial of Catheter Ablation Versus Medical Treatment of Atrial Fibrillation in Heart Failure; **CASTLE-AF:** Catheter Ablation versus Standard Conventional Therapy in Patients with Left Ventricular Dysfunction and Atrial Fibrillation; **CI:** Confidence Interval; **CV:** Cardiovascular; **HF:** Heart Failure; **LVEF:** Left Ventricular Ejection Fraction; **MLHFQ:** Minnesota Living with Heart Failure Questionnaires; **SD:** Standard Deviation; **RAFT:** Randomized Ablation-Based Rhythm-Control Versus Rate-Control.*

Supplemental Figure S5. Catheter Ablation vs Drug Therapy in patients with Persistent AF. Forest plots displaying mean differences in all-cause mortality (a), HF hospitalization (b), CV death (c), AF recurrence (d), LVEF (e), 6MWT (f) and MLFHQ (g) between ablation and drug therapy in patients with persistent AF.

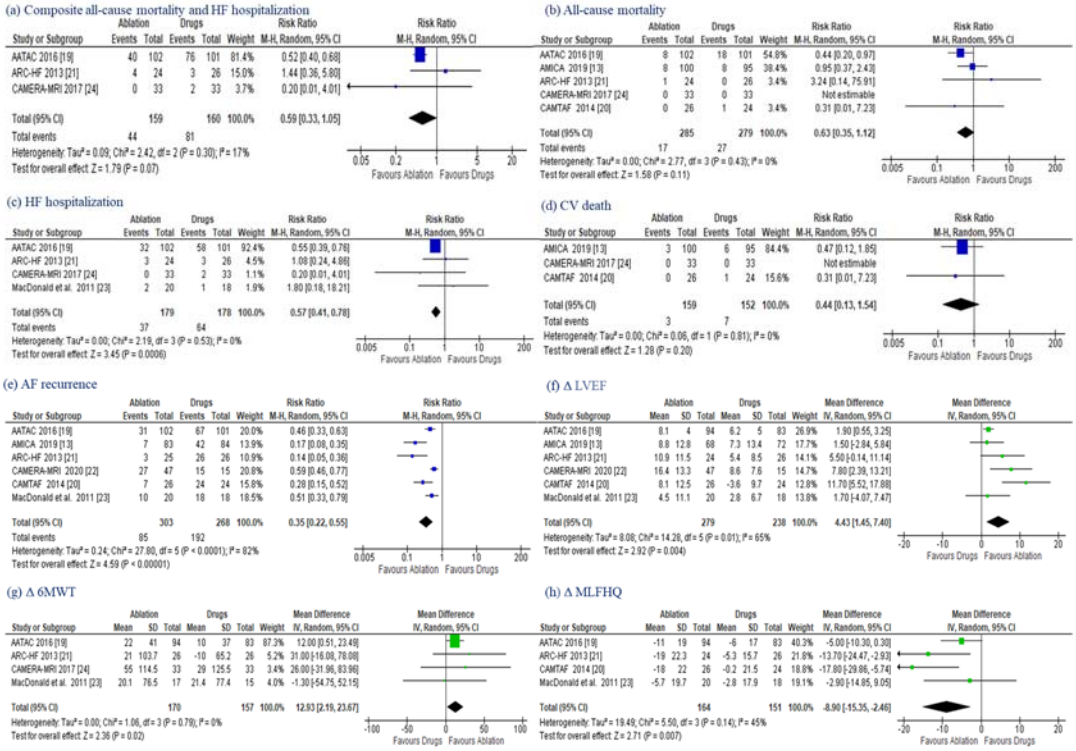

***6MWT:** 6 Minute Walk Test; **AF:** Atrial Fibrillation; **AATAC:** Ablation versus Amiodarone for Treatment of Atrial Fibrillation in Patients with Congestive Heart Failure and an Implanted ICD; **AMICA:** Atrial Fibrillation Management in Congestive Heart Failure With Ablation; **ARC-HF:** A Randomized Trial to Assess Catheter Ablation Versus Rate Control in the Management of Persistent Atrial Fibrillation in Chronic Heart Failure; **CAMERA-MRI:** Catheter Ablation Versus Medical Rate Control in Atrial Fibrillation and Systolic Dysfunction; **CAMTAF:** A Randomized Controlled Trial of Catheter Ablation Versus Medical Treatment of Atrial Fibrillation in Heart Failure; **CI:** Confidence Interval; **CV:** Cardiovascular; **HF:** Heart Failure; **LVEF:** Left Ventricular Ejection Fraction; **MLHFQ:** Minnesota Living with Heart Failure Questionnaires; **SD:** Standard Deviation; **RAFT:** Randomized Ablation-Based Rhythm-Control Versus Rate-Control.*
